# Genomic alterations during p53-dependent apoptosis induced by γ-irradiation of Molt-4 leukemia cells

**DOI:** 10.1371/journal.pone.0190221

**Published:** 2017-12-22

**Authors:** Rouba Hage-Sleiman, Hisham Bahmad, Hadile Kobeissy, Zeinab Dakdouk, Firas Kobeissy, Ghassan Dbaibo

**Affiliations:** 1 Department of Biology, Faculty of Sciences, Lebanese University, Hadath, Lebanon; 2 Department of Anatomy, Cell Biology and Physiological Sciences, Faculty of Medicine, American University of Beirut, Beirut, Lebanon; 3 Department of Biochemistry and Molecular Genetics, Faculty of Medicine, American University of Beirut, Beirut, Lebanon; 4 Department of Pediatrics and Adolescent Medicine, Center for Infectious Diseases Research, Faculty of Medicine, American University of Beirut, Beirut, Lebanon; Virginia Commonwealth University, UNITED STATES

## Abstract

Molt-4 leukemia cells undergo p53-dependent apoptosis accompanied by accumulation of *de novo* ceramide after 14 hours of γ-irradiation. In order to identify the potential mediators involved in ceramide accumulation and the cell death response, differentially expressed genes were identified by Affymetrix Microarray Analysis. Molt-4-LXSN cells, expressing wild type p53, and p53-deficient Molt-4-E6 cells were irradiated and harvested at 3 and 8 hours post-irradiation. Human genome U133 plus 2.0 array containing >47,000 transcripts was used for gene expression profiling. From over 10,000 probes, 281 and 12 probes were differentially expressed in Molt-4-LXSN and Molt-4-E6 cells, respectively. Data analysis revealed 63 (upregulated) and 20 (downregulated) genes (>2 fold) in Molt-4-LXSN at 3 hours and 140 (upregulated) and 21 (downregulated) at 8 hours post-irradiation. In Molt-4-E6 cells, 5 (upregulated) genes each were found at 3 hours and 8 hours, respectively. In Molt-4-LXSN cells, a significant fraction of the genes with altered expression at 3 hours were found to be involved in apoptosis signaling pathway (*BCL2L11*), p53 pathway (*PMAIP1*, *CDKN1A* and *FAS*) and oxidative stress response (*FDXR*, *CROT* and *JUN*). Similarly, at 8 hours the genes with altered expression were involved in the apoptosis signaling pathway (*BAX*, *BIK* and *JUN*), p53 pathway (*BAX*, *CDKN1A* and *FAS*), oxidative stress response (*FDXR* and *CROT*) and p53 pathway feedback loops 2 (*MDM2* and *CDKN1A*). A global molecular and biological interaction map analysis showed an association of these altered genes with apoptosis, senescence, DNA damage, oxidative stress, cell cycle arrest and caspase activation. In a targeted study, activation of apoptosis correlated with changes in gene expression of some of the above genes and revealed sequential activation of both intrinsic and extrinsic apoptotic pathways that precede ceramide accumulation and subsequent execution of apoptosis. One or more of these altered genes may be involved in p53-dependent ceramide accumulation.

## Introduction

Chemotherapeutics [1] and irradiation [2–3] are genotoxic stressors widely used in the treatment of various cancers and known to stimulate cell cycle arrest or apoptosis depending on the cell type. The tumor suppressor p53 plays a central role in the response to genotoxic stress and is critical in mediating apoptosis during cancer therapy. Mutation of p53, common in cancers that have already been exposed to chemotherapeutic agents, is a cause of chemoresistance and failure of cancer therapy [4]. Thus, there is a great interest in understanding the pathways activated by p53 in order to identify potential alternatives for targeted therapy in cancers where p53 has mutated.

The sphingolipid ceramide is emerging as an important bioactive lipid in cancer treatment [5]. Ceramide, capable of inducing apoptosis and cell cycle arrest in a wide variety of cancer and normal cell types, may accumulate following exposure to γ-irradiation or chemotherapeutic agents, in both p53-dependent and p53-independent manners [6–8]. Importantly, ceramide can induce apoptosis equally well in cancers that have or lack functional p53 [8]. Molt-4 leukemia cells undergo p53-dependent ceramide accumulation and cell death upon exposure to actinomycin D or γ-irradiation [8]. The observed ceramide accumulation was attributed to *de novo* ceramide biosynthesis by the activation of ceramide synthase, specifically CerS5, responsible for C16 ceramide generation [9]. Although minimal transcriptional upregulation of CerS5 was observed in Molt-4 cells, it was not detectable in another p53-dependent colon cancer cell model [9]. Moreover, ceramide still accumulated in a p53-dependent manner in the presence of cycloheximide indicating that it can also be generated in a transcriptionally-independent manner [10]. Therefore, ceramide generated downstream of p53 presents an interesting therapeutic target in p53-deficient cancer cells as it may play an important role in p53-independent apoptosis.

In order to identify the potential pathways activated by p53 that induce ceramide accumulation and apoptosis in Molt-4 cells, differentially expressed genes that are potential mediators were identified by Affymetrix Microarray Analysis in irradiated Molt-4-LXSN (wild-type p53) as compared to p53-deficient Molt-4-E6 cells at time points that precede any detectable ceramide accumulation and cell death. Human genome U133 plus 2.0 array, which contains >47,000 transcripts, was used for gene expression profiling. Over 10,000 probes, 12 probes were differentially expressed in Molt-4-E6 cells and 281 probes in Molt-4-LXSN cells. Some differentially modified targets were validated at the protein level and the pathways involved in the response of Molt-4 cells to irradiation were also identified.

## Materials and methods

### Cell lines and culture

Human leukemia cell line Molt-4 was obtained from American Type Culture Collection. Molt-4-LXSN cells (stably transfected with empty retroviral vector LXSN) and Molt-4-E6 (stably transfected with the human papillomavirus E6 gene cloned into the LXSN retroviral vector, a kind gift from Dr. Denise Galloway, University of Washington, Seattle, WA) were described previously [11, 8]. Stable transfectants were grown in RPMI 1640 supplemented with 10% FBS and 1% penicillin-streptomycin, and maintained by selection with 500 μg/ml geneticin (G418). Experiments were done in the absence of G418.

### RNA extraction

Molt-4-LXSN and Molt-4-E6 cells were irradiated with 5 Gy of γ rays using a Cesium source ^137^Cs (2441.1cGy/min). Non-irradiated controls and irradiated cells (harvested 3 and 8 hours post-irradiation) were pelleted and total RNA was extracted using the RNeasy Plus mini kit (Qiagen) according to the manufacturer’s recommendations. Total RNA yield and purity were determined using Nanodrop ND1000 (Thermo Fisher Scientific) and RNA integrity was validated using 1% agarose gel electrophoresis. Three independent experiments were performed.

### Affymetrix gene expression profiling

Human genome U133 plus 2.0 arrays (Affymetrix, Santa Clara, CA), which contain >47,000 transcripts, were used for the gene expression profiling of control and irradiated Molt-4-LXSN and Molt-4-E6 cells of three independent experiments. Double-stranded cDNA was synthesized from 10 μg of total RNA using the one-cycle cDNA synthesis kit; subsequently, biotin-labeled anti-sense cRNA was synthesized using the *in vitro* transcription labeling kit (Affymetrix) and fragmented. A hybridization cocktail containing the fragmented cRNA, probe array controls (Affymetrix), bovine serum albumin (BSA), and herring sperm DNA (Life Technologies, Grand Island, NY) was hybridized to human genome U133 plus 2.0 arrays for 16 hours. Hybridization, washing, and staining of the arrays were performed on a Fluidics station (Affymetrix); all protocols were performed in accordance with the Affymetrix gene expression profiling technical manual. After hybridization and scanning, the data were analyzed using Bioconductor packages (http://www.bioconductor.org) within the open source R statistical environment (http://www.r-project.org). The quality control metrics recommended by Affymetrix, box plots, and intensity histograms were used for quality assessment. After background correction by robust multiarray analysis, a filter using the SD of gene expression values was applied to select the top most variable 10,000 genes. For differential expression analysis, Limma was used [12]. A double cutoff of false-discovery rate <0.05 and a fold change of two or greater was applied.

### Western blot

Protein expression levels were analyzed using 12 and 15% acrylamide gels. Samples were prepared with a 1:1 volume ratio of proteins to loading buffer [Tris-HCl 0.25 M (pH 6.8), 4% SDS, 20% Glycerol, 2 mg bromophenol blue, and 5% β-mercaptoethanol] and run using TGS 1X running buffer [TGS 10X: 30 g Tris (hydroxymethyl)-aminomethane, 144 g glycine and 10 g SDS]. The migration was performed at 80 V for the stacking gel and 120 V for the resolving gel. Following migration, transfer to a polyvinylidene difluoride (PVDF) membrane was done in transfer buffer [TGS 1X with 20% methanol] for 90 min at 100 V. Then, the membrane was blocked to prevent nonspecific binding using 5% fat-free milk prepared in TBS 1X [TBS 10X: 12 g Tris (hydroxymethyl)-aminomethane and 87.8 g NaCl, pH.8] with 0.1% Tween for 2 hours. Following blocking, the membrane was incubated at 4°C overnight with 2 ml of specific primary antibody diluted in 5% milk-TBS 1X 0.1% Tween as recommended by the supplier. The membrane was then washed for 10 minutes with TBS 1X 0.1% Tween for three cycles and incubated at room temperature for one hour with 5 ml of the horseradish peroxidase (HRP)-conjugated secondary antibodies (Jackson ImmunoResearch, Europe) diluted in 5% milk- TBS 1X 0.1% Tween as recommended by the supplier. Finally, the bands were developed using ECL western blotting reagent (GE health care, UK). GAPDH (sc-47724) was used as loading control. Antibodies against human Fas (C-20; sc-715), Noxa (Calbiochem; 140129), Bax (B-9; sc-7480), Bim (Y-36; ab32158), Cyclin B1 (H-433; sc-752) and p21 (F-5; sc-6246) were used in this study. Densitometric quantification of western blots was performed by ImageJ software (NIH, USA) and the statistical significance was determined with a Student's t-test.

### Venn diagram overlap of individual gene lists

Differentially expressed gene sets in Molt-4-LXSN and Molt-4-E6 cells were analyzed using Venny, a web-based tool. http://bioinfogp.cnb.csic.es/tools/venny/index.html

### Biological pathways, systems biology analysis and statistical testing

The microarray differential expression of the Molt-4-LXSN experimental condition at the 3 hours and 8 hours post-irradiation versus non-irradiated control Molt-4-LXSN was further analyzed using a systems biology approach assessing altered pathways contributing to the p53-dependent apoptosis induced by irradiation. The Elsevier’s PathwayStudio version 10.0 (Ariadne Genomics/Elsevier, Rockville, MD, USA) was used to assess and analyze relationships between the differentially expressed gene candidates using the Ariadne ResNet database [13–14]. Statistically significant altered pathways pertaining to each identified gene set of the Molt-4-LXSN/controls groups were extracted via “Subnetwork Enrichment Analysis” (SNEA) algorithm assessing global and targeted analysis on the altered gene sets. PathwayStudio utilizes a built-in resource ResNet database, which extracts molecular interactions based on natural language processing of scientific abstracts in PubMed. SNEA utilizes Fisher's statistical test used to determine if there are nonrandom associations between two categorical variables organized by specific relationship. Global and targeted analysis was performed on the altered gene sets. For the comparative analysis of the altered enriched pathways among the Molt-4-LXSN/controls groups, “InteractiVenn” software,a web-based tool was used to analyze complex data sets [15].

### Heat map generation

Differentially expressed genes were hierarchically clustered using average linkage based on the Pearson correlation coefficient that was calculated using normalized scaled log2 intensity levels. The heat map was created using the cluster 3.0 and TreeView 3.0 softwares.

### Gene ontology and biological process

To better interpret the heat map clustering, Biological Process extracted from gene ontology analysis was assessed to depict how these genes cluster in their corresponding statistically significant biological process. For this analysis, PANTHER bioinformatics software (Protein ANalysis THrough Evolutionary Relationships; http://www.pantherdb.org/genes/batchIdSearch.jsp) was used. PANTHER software classifies gene into distinct categories of molecular functions, biological processes and cellular localization. It relies on published scientific experimental evidence and evolutionary relationships from the GO database which are curated for function and process prediction. Proteins are classified into families and subfamilies of shared function, which are then categorized using a highly controlled vocabulary (ontology terms) by biological process, molecular function and molecular pathway.

## Results

### Hierarchical clustering of genes in response to γ-irradiation

**Fig 1:**
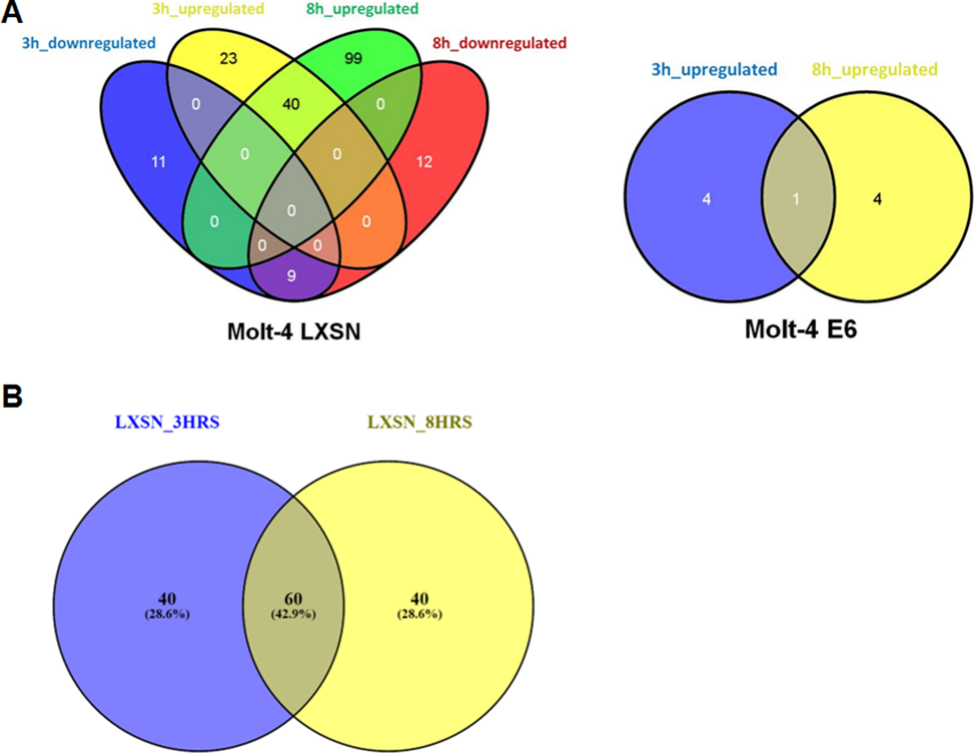
Venn diagram analysis of altered genes and biological pathways. **(A)** Venn diagram showing the number of common and unique genes, upregulated and downregulated, in irradiated Molt-4- LXSN (S1 and S2 Tables) and Molt-4-E6 (S3 and S4 Tables) cells at 3hrs and 8hrs as compared to non-irradiated cells. **(B)** Venn diagram showing the number of common and unique pathways detected in irradiated Molt-4- LXSN cells at 3hrs and 8hrs as compared to non-irradiated cells. (S5 Table).

**Fig 2:**
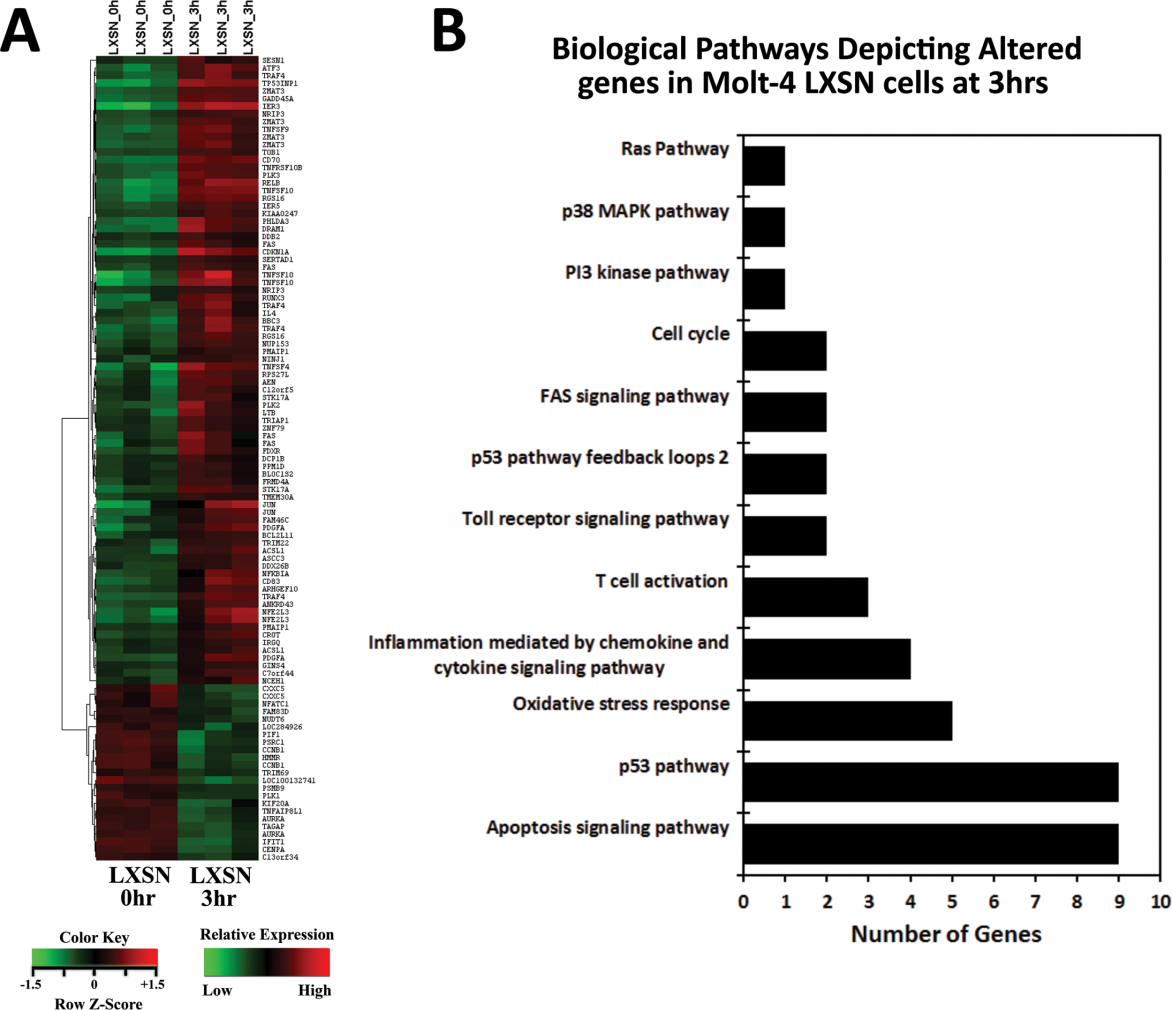
Microarray analysis of altered genes in irradiated Molt-4-LXSN cells at 3hrs vs. 0hr. **(A)** Heat map from hierarchical clustering analysis of the differentially expressed genes. Each column represents one sample, and each row refers to a gene. Color legend is represented on the figure. Red indicates genes with higher expression relative to the geometrical means; green indicates genes with lower expression relative to the geometrical means (S6 Table). **(B)** Biological Process Gene Ontology (GO) analysis of the differentially expressed genes at 3hrs classified into 12 categories, many of which share the same genes according to their functional correlation (S7 Table).

**Fig 3:**
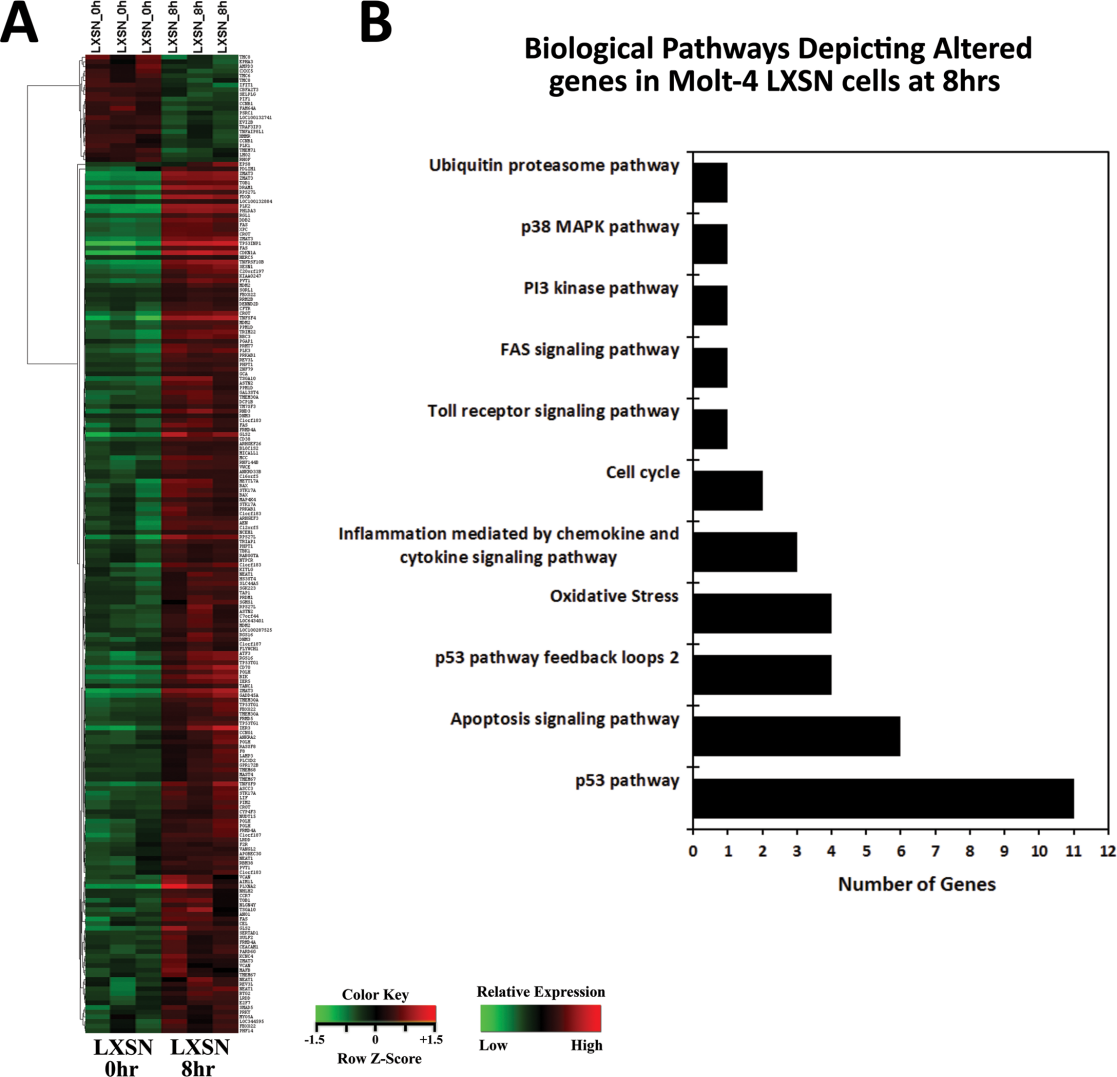
Microarray analysis of altered genes in irradiated Molt-4-LXSN cells at 8hrs vs. 0hr. **(A)** Heat map from hierarchical clustering analysis of the differentially expressed genes. Each column represents one sample, and each row refers to a gene. Color legend is represented on the figure. Red indicates genes with higher expression relative to the geometrical means; green indicates genes with lower expression relative to the geometrical means (S6 Table). **(B)** Biological Process Gene Ontology (GO) analysis of the differentially expressed genes at 8hrs classified into 11 categories, many of which share the same genes according to their functional correlation (S8 Table).

Upon γ-irradiation (5Gy) of Molt-4 cells, 12 probes were differentially expressed in Molt-4-E6 cells and 281 probes in Molt-4-LXSN cells. Data analysis revealed 63 (upregulated) and 20 (downregulated) genes in Molt-4-LXSN at 3 hours and 139 (upregulated) and 21 (downregulated) at 8 hours post-irradiation (Fig 1A). Venn diagram analysis showed that of the 63 upregulated genes at 3 hours, 40 remained upregulated at 8 hours and of the 20 downregulated at 3 hours, 9 remained downregulated at 8 hours (Fig 1A). In Molt-4-E6 cells, 5 (upregulated) and 5 (upregulated) genes were found at 3 hours and 8 hours, respectively, with only a single gene (*LTB*, lymphotoxin beta) having sustained upregulation at both 3 and 8 hours (Fig 1A). All the differentially expressed (DE) genes in Molt-4-LXSN and Molt-4-E6 cells are listed with fold change (FC) values in the S1 to S4 Tables. Venn diagram analysis of altered biological pathways in Molt-4-LXSN cells identified unique statistically significant pathways including 40 pathways (3hrs vs. 0hr) and 40 pathways (8hrs vs. 0hr) (Fig 1B). Common statistically significant pathways were identified including 60 pathways (3hrs vs. 8hrs vs. 0hr) (Fig 1B). The detailed lists of common and unique pathways are available in the S5 Table. A heat map hierarchical clustering of gene expression was performed for Molt-4-LXSN at 3 hours (Fig 2A) and 8 hours (Fig 3A) post-irradiation, based on the differential expression level (2-fold up or down), and visualized using a color scale.

### Analysis of biological pathways in response to γ-irradiation

Biological Process Gene Ontology (GO) analysis of the differentially expressed genes was done on Molt-4-LXSN at 3 hours and 8 hours post-irradiation and showed a classification into 12 and 11 categories, respectively. Many biological processes share the same genes according to their functional correlation. We found an over-representation of themes related to apoptosis signaling pathway (22% and 31%), p53 pathway (22% and 17%), and oxidative stress response (12% and 11%) in Molt-4-LXSN cells after 3 hours (Fig 2B) and 8 hours (Fig 3B) of irradiation, respectively. It is not surprising that p53 signaling pathway was the most represented pathway in our model. On the contrary, this supports the previous study done on this model [8] that suggests a ceramide and p53-dependent cell death response to γ-irradiation. Other biological pathways included inflammation mediated by chemokine and cytokine signaling pathway, T cell activation, Toll receptor signaling pathway, FAS signaling pathway, PI3 kinase pathway, p38 MAPK pathway, cell cycle, ubiquitin proteasome pathway, p53 pathway feedback loops 1 and 2. The relations of altered genes to different pathways are detailed in the S7 and S8 Tables.

### Altered genes in response to γ-irradiation

**Fig 4:**
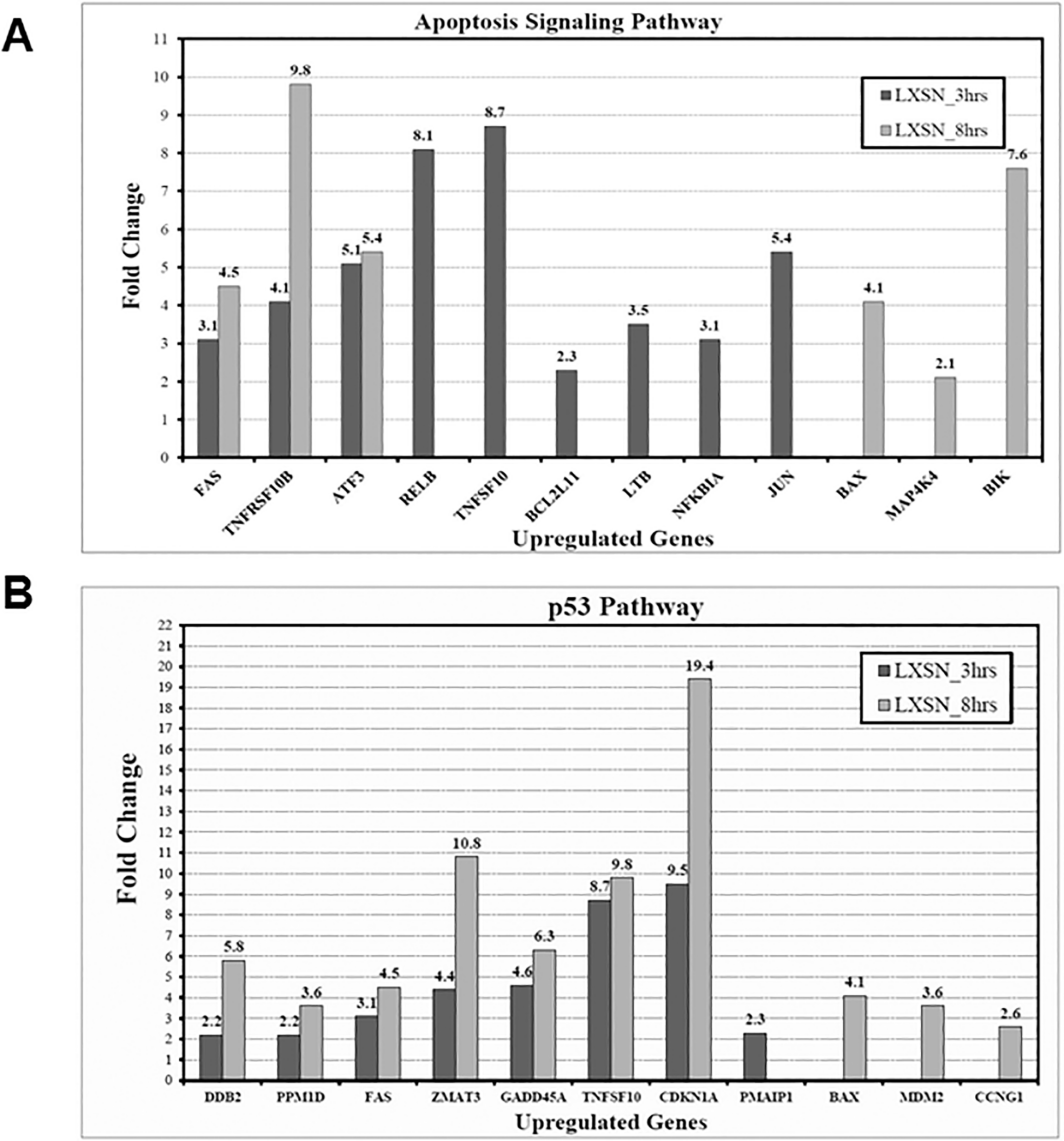
Bar graph depicting the Fold Changes (FCs) of differentially upregulated genes of the **(A)** apoptosis signaling pathway and **(B)** p53 pathway in Molt-4-LXSN cells at 3hrs and 8hrs as compared to non-irradiated cells.

Differentially expressed genes in Molt-4-LXSN cells in response to γ-irradiation were categorized according to their biological pathways and presented in Tables 1 and 2. All the cited genes were upregulated except for *CCNB1/cyclin B1* (FC = -2.74 and -2.31 at 3 hours and 8 hours, respectively). Since irradiated Molt-4-LXSN cells represent a model of p53-dependent cell death, we were interested in two pathways, apoptosis signaling pathways and p53 pathway, which also turned to be the most represented ones. The apoptosis signaling (Fig 4A) and p53 (Fig 4B) pathways related genes *Bim/BCL2L11* (FC = 2.3), *Fas* (FC = 3.1) and *PMAIP1/Noxa* (FC = 2.27) were upregulated after 3 hours of irradiation. Cell cycle related gene *CDKN1A/p21* (FC = 9.5) was upregulated after 3 hours of irradiation. After 8 hours of irradiation, *Bax* (FC = 4.06), *Bik* (FC = 7.63), *CDKN1A/p21* (FC = 19.36) and *Fas* (FC = 4.53) were upregulated. Many other genes such as *Mdm2*, *ATF3*, *Jun* and *TNFSF10* were also significantly upregulated. We performed western blots to correlate the observed alterations in the mRNA levels of these latter genes with their relative protein levels.

**Table 1:** Biological pathways Gene Ontology (GO) analysis of differentially expressed genes in Molt-4-LXSN cells at 3hrs vs. 0hr. Many categories shared the same transcripts.

Term	Genes Count	Overlapping Entities
Apoptosis signaling pathway (P00006)	9	*RELB*, *TNFSF10*, *TNFRSF10B*, *BCL2L11*, *LTB*, *NFKBIA*, *ATF3*, *FAS*, *JUN*
p53 pathway (P00059)	9	*PMAIP1*, *TNFSF10*, *CCNB1*, *PPM1D*, *GADD45A*, *ZMAT3*, *CDKN1A*, *FAS*, *DDB2*
Inflammation mediated by chemokine and cytokine signaling pathway (P00031)	4	*RELB*, *NFATC1*, *NFKBIA*, *JUN*
Gonadotropin releasing hormone receptor pathway (P06664)	3	*NFATC1*, *ATF3*, *JUN*
B cell activation (P00010)	3	*NFATC1*, *NFKBIA*, *JUN*
CCKR signaling map (P06959)	3	*IER3*, *NFKBIA*, *JUN*
T cell activation (P00053)	3	*NFATC1*, *NFKBIA*, *JUN*
Angiogenesis (P00005)	2	*PDGFA*, *JUN*
Interleukin signaling pathway (P00036)	2	*CDKN1A*, *IL4*
p53 pathway feedback loops 2 (P04398)	2	*PPM1D*, *CDKN1A*
PDGF signaling pathway (P00047)	2	*PDGFA*, *JUN*
Toll receptor signaling pathway (P00054)	2	*NFKBIA*, *JUN*
TGF-beta signaling pathway (P00052)	2	*DCP1B*, *JUN*
FAS signaling pathway (P00020)	2	*FAS*, *JUN*
p38 MAPK pathway (P05918)	1	*GADD45A*
Vitamin D metabolism and pathway (P04396)	1	*FDXR*
PI3 kinase pathway (P00048)	1	*GADD45A*
Oxidative stress response (P00046)	1	*JUN*
Ras Pathway (P04393)	1	*JUN*
Cell cycle (P00013)	1	*CCNB1*
Huntington disease (P00029)	1	*JUN*
Heterotrimeric G-protein signaling pathway-Gq alpha and Go alpha mediated pathway (P00027)	1	*RGS16*
Wnt signaling pathway (P00057)	1	*NFATC1*
Heterotrimeric G-protein signaling pathway-Gi alpha and Gs alpha mediated pathway (P00026)	1	*RGS16*

**Table 2:** Biological pathways Gene Ontology (GO) analysis of differentially expressed genes in Molt-4-LXSN cells at 8hrs vs. 0hr. Many categories shared the same transcripts.

Term	Genes Count	Overlapping Entities
Apoptosis signaling pathway (P00006)	6	*BAX*, *TNFRSF10B*, *MAP4K4*, *BIK*, *ATF3*, *FAS*
p53 pathway (P00059)	11	*BAX*, *TNFRSF10B*, *CCNB1*, *PPM1D*, *MDM2*, *GADD45A*, *ZMAT3*, *CDKN1A*, *FAS*, *DDB2*, *CCNG1*
Inflammation mediated by chemokine and cytokine signaling pathway (P00031)	3	*XPC*, *PRKY*, *CCR7*
Toll receptor signaling pathway (P00054)	1	*TBK1*
p53 pathway feedback loops 2 (P04398)	4	*PPM1D*, *MDM2*, *CDKN1A*, *CCNG1*
FAS signaling pathway (P00020)	1	*FAS*
PI3 kinase pathway (P00048)	1	*GADD45A*
p38 MAPK pathway (P05918)	1	*GADD45A*
Cell cycle (P00013)	1	*CCNB1*
Ubiquitin proteasome pathway (P00060)	1	*MDM2*
p53 pathway feedback loops 1 (P04392)	1	*MDM2*

### Validation of selected candidate genes

**Fig 5:**
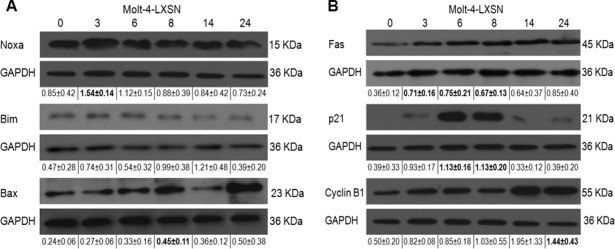
Protein expression levels of some altered genes in response to irradiation. Western blot analysis of the expression level of **(A)** Noxa, Bim, and Bax and **(B)** Fas, p21, and Cyclin B1 was assayed on total lysates of Molt-4-LXSN cells after 3, 6, 8, 14 and 24 hours post-irradiation. Each blot is representative of three independent experiments. GAPDH was used as loading control in membranes. Quantification of band intensity was performed by ImageJ. Values below each blot represent the average ± standard deviation of three independent experiments. Bold values represent significant difference (P < 0.05) with respect to non-irradiated cells.

Microarray analysis revealed the upregulation of *Noxa*, *Bim/BCL2L11*, *CDKN1A/p21* and *Fas* and downregulation of *CCNB1/cyclin B1* at 3 hours post-irradiation. Western blotting for Noxa showed higher levels of the protein at 3 hours and 6 hours post-irradiation. Densitometric analysis of the bands revealed a significant upregulation of Noxa (1.8 fold increase) at 3 hours as compared to non-irradiated cells and normalized to GAPDH expression level (Fig 5A). Results of Bim showed an increase in protein expression starting 3 hours till 8 hours but without any statistically significant difference (Fig 5A). Protein expression levels of Fas significantly increased from 3 hours till 8 hours post-irradiation and remained high at 14 and 24 hours (Fig 5B). As for p21, it showed higher levels of the protein at 3, 6 and 8 hours however it only reached significance at 6 and 8 hours post-irradiation (Fig 5B). Validation of *Bax* upregulation on the protein level showed a significant upregulation at 8 hours post-irradiation (1.9 fold increase) (Fig 5A). Finally, the only selected candidate downregulated in the microarray analysis, cyclin B1, was studied by western blot. Results showed an increase in the expression levels of the protein. However this increase was not significant and stabilized between 3 and 6 hours (Fig 5B).

### Systems biology analysis of altered genes in response to γ-irradiation

**Fig 6:**
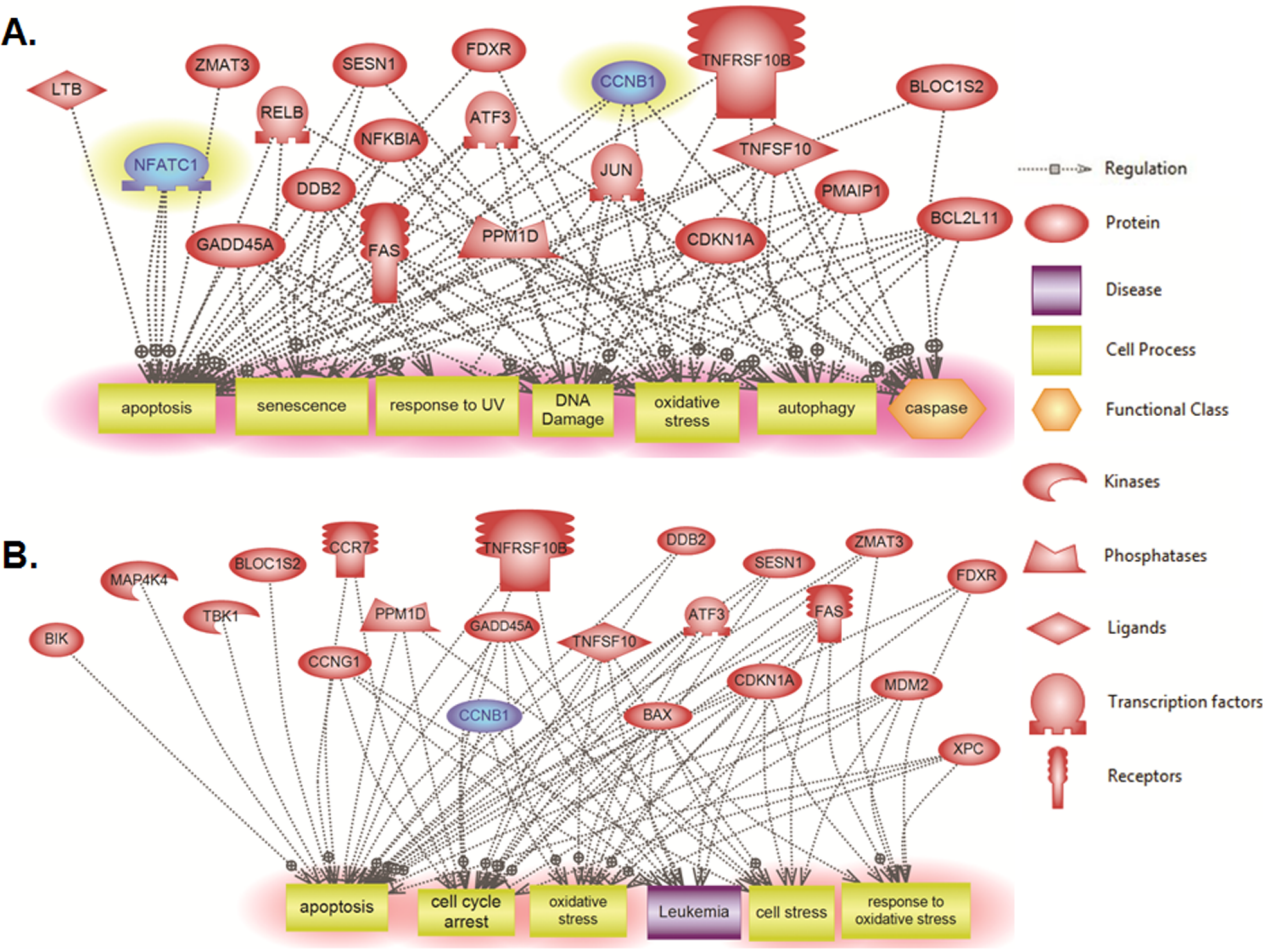
Global molecular and biological pathways’ interaction map analysis in Molt-4- LXSN cells. Using Pathway Studio 10.0, altered genes relevant to γ-irradiation in Molt-4-LXSN cells were analyzed at **(A)** 3 hours (S9 Table) and **(B)** 8 hours (S10 Table) post-irradiation. “Direct interaction” algorithm was used to generate and map the global network of biological processes and interactions among altered genes. The upregulated genes are shown in light red and downregulated genes are in blue. The shape of each given protein is indicative of its functional class as shown in the legend.

**Fig 7:**
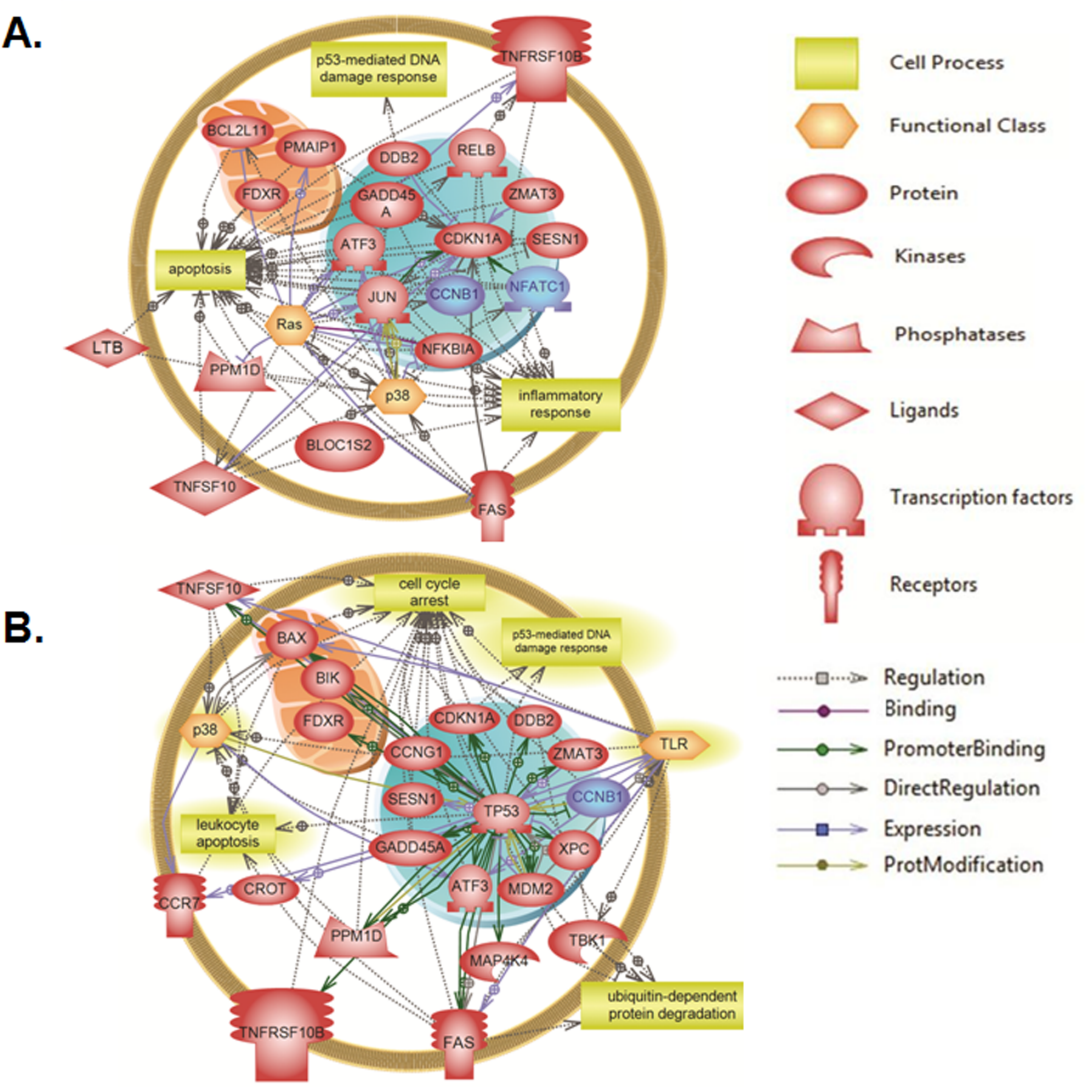
Targeted molecular and biological pathways’ interaction map analysis in Molt-4-LXSN cells. Using Pathway Studio 10.0, altered genes relevant to γ-irradiation in Molt-4-LXSN cells were analyzed at **(A)** 3 hours (S11 Table) and **(B)** 8 hours (S12 Table) post-irradiation. “Direct interaction” algorithm was used to generate and map the targeted network of biological processes and interactions among altered genes. The upregulated genes are shown in light red and downregulated genes are in blue. Each of the proteins is displayed within a subcellular compartment or organelle. The shape of each given protein is indicative of its functional class as shown in the legend. Included in the legend is the definition of the lines connecting 2 genes.

Elsevier’s Pathway Studio 10.0 (Ariadne Genomics/Elsevier) was also used to generate and map the network of biological processes and interactions among altered genes. Results of global molecular and biological pathways analysis for Molt-4-LXSN cells at 3 hours revealed association of the altered genes mainly with apoptosis, DNA damage, oxidative stress and caspase, along with alterations in the genes related to senescence and response to UV (Fig 6A). The results at 8 hours post-irradiation showed additional biological processes such as cell cycle arrest, cell stress and response to oxidative stress along with marked alterations in the genes related to leukemia pathways (Fig 6B). A targeted molecular and biological pathways analysis was also performed in order to determine the relatedness of the altered genes at 3 hours post-irradiation to apoptosis, inflammatory response and p53-mediated DNA damage response (Fig 7A). Altered genes at 8 hours were linked to leukocyte apoptosis, cell cycle arrest and ubiquitin-dependent protein degradation (Fig 7B). Furthermore, the targeted systems biology analysis illustrated a role for the Ras and p38 signaling pathways in the responses associated with the altered genes at 3 hours and 8 hours upon irradiation. TP53 appears essential to many of the altered genes and is required for all of the responses at 8 hours post-irradiation. The altered genes localize to the nucleus (*CCNB1/cyclin B1*, *Jun*, *Mdm2*, *ATF3* and *CDKN1A/p21*), the mitochondria (*Bim/BCL2L11*, *PMAIP1/Noxa*, *Bax* and *Bik*) and cell membrane (*Fas*, *TNFRSF10B* and *CCR7*).

## Discussion

Studies have shown that p53-dependent apoptosis induced by γ-irradiation is regulated by Bcl-2 family proteins [16] and mediated by p53-dependent cleavage of caspase-8 and caspase-9 in Molt-4 cells upon γ-irradiation or x-irradiation [17–18]. Exposure of Molt-4-LXSN leukemia cells to γ-irradiation induces p53 upregulation after 2 hours followed by ceramide accumulation at 14 hours and cell death detectable by 24 hours [8]. The p53-deficient Molt-4-E6 cells were resistant to γ-irradiation [8]. Upon p53 upregulation, ceramide is generated through *de novo* ceramide synthesis, specifically associated with ceramide synthase 5, rather than serine palmitoyltransferase, activity [9]. In the current study, neither sphingolipid regulating genes nor sphingolipid enzymes were found differentially modulated. This suggests that in response to irradiation, a post-transcriptional response leading to ceramide synthesis is triggered and that transcriptional regulation of this pathway is not involved. This supports the finding that *de novo* ceramide synthesis is regulated by a rapid post-translational mechanism involving the dimerization of ceramide synthase [19]. In the endoplasmic reticulum, ceramide synthase exists in equilibrium between monomers and dimers, and its quick dimerization is a major way of regulating its activity and increasing ceramide synthesis under various physiological conditions [19].

In this study, we present evidence that γ-irradiation stimulates genes involved in both the intrinsic and extrinsic pathways of apoptosis. The extrinsic pathway is mediated by cell surface death receptors, including Fas, TNF, and DR4/DR5 receptors. Ligand stimulation of the death receptors induces the clustering of the death domains and the subsequent formation of death inducing signaling complex (DISC), which then leads to activation of caspase-8 and subsequently the activation of caspase-3 [20]. We found that *Fas* and *TNFRSF10B* death receptors are upregulated differentially only in the Molt-4-LXSN and not in the Molt-4-E6 cells indicating that their transcription was p53-dependent [21]. The increase in Fas protein level was validated after 3 hours and 8 hours post-irradiation. The significance of their upregulation in the absence of corresponding upregulation of their ligands is unclear, but there is evidence that the death receptors may initiate apoptosis independently of their respective ligands by induction of clustering and resulting autoactivation [22]. Alternatively, this may serve to prime the cells for extrinsic apoptosis induced by other immune cells that express or secrete their respective ligands. Ceramide is one of the mediators that potentiate receptors’ oligomerization by forming lipid rafts in the plasma membrane. In fact, activation of caspase-8 in the extrinsic apoptosis causes the translocation of acid sphingomyelinase from the lysosomes to the plasma membrane where sphingomyelin is hydrolyzed into ceramide [23]. Thus, ceramide generated during p53-dependent apoptosis may serve as a facilitator of death receptor-induced signaling at the plasma membrane.

The intrinsic pathway is activated at the mitochondrial level as a result of cellular stress and DNA damage [24]. In this pathway, the Bcl-2 family of proteins are the most important regulators [24]. The pro-apoptotic members are divided into two groups: 1) the BH3-only proteins (Noxa, Puma, Bid, Bad, and Bim) and 2) the effector or executioner proteins (Bax and Bak) [25], which contribute to the permeabilization of the outer mitochondrial membrane and the release of apoptotic factors such as cytochrome c, and Smac/Diablo into the cytosol [24]. Cytosolic cytochrome c activates the apoptotic protease activating factor (apaf-1) and induces the formation of the apoptosome [26], which activates caspase-9. In the same context, activated caspase-9 in the apoptosome, in turn, activates the effector caspases-3, 6, and 7 by cleavage of their respective pro-caspases [27]. On the other hand, the anti-apoptotic Bcl-2 proteins (Bcl-2, Mcl-1 and Bcl-xL) are located on either cellular membranes or in the cytosol and inhibit the oligomerization of the effector proteins Bax and Bak [28]. Upon γ-irradiation, many Bcl-2 proteins relevant to the intrinsic pathway of apoptosis were found to be differentially regulated in Molt-4-LXSN cells. *Noxa*, *Bim* and *Bax* were upregulated in response to p53 induction in our model. *Noxa* is transcriptionally activated by p53 [29] and its protein product translocates to the mitochondria where it sensitizes for, rather than activates, cell death. It is Bax and Bak that act as executioners of cell death and oligomerize on the mitochondrial membrane along with ceramide molecules, permeabilizing it and causing the release of cytochrome c [30].

The nuclear factor kappa B (NF-κB) is an important transcriptional regulator of pro-inflammatory cytokines, cytokine receptors, and anti-apoptotic proteins [31]. Inhibitor of kappa B protein (IκB) binds NF-κB in the cytosol and prevents its release to the nucleus until it is phosphorylated by various kinases, including protein kinase C (PKC) isoforms, which leads to its proteasomal degradation [32]. In the current study, *IκBα*/*NFKBIA* was upregulated at 3 hours post-irradiation in Molt-4-LXSN cells. This suggests a potential repression or limitation of the anti-apoptotic proteins regulated by NF-κB, thus favoring apoptosis. Interestingly, ceramide has been shown to inhibit NF-κB activation by inhibiting PKC [33]. These findings indicate that p53 and ceramide may cooperate to create a favorable pro-apoptotic milieu.

A recent review paper investigated 3,509 candidate p53 target genes derived from 16 high-throughput data sets and reported that *Fas*, *Bax* and *Noxa* were identified in at least 6 out 16 genome wide-data sets [34]. Interestingly, *p21* was identified in the 16 data sets [34]. In our study, cell cycle arrest was among the biological pathways in addition to apoptosis, oxidative stress, and DNA damage. Our results revealed an important role of cell cycle related genes such as *p21* and *cyclin B1* shown to be upregulated and downregulated, respectively at 3 and 8 hours post-irradiation. The cyclin-dependent kinase (CDK) inhibitor p21/CDKN1A is necessary for p53-dependent downregulation of cell cycle regulating genes *CDK1*, *Cyclin A2* [35–36] and *cyclin B* [37]. Here we suggest a possible explanation for the role of cell cycle related genes in the cell response to irradiation. Under genotoxic stress, p53 induces G2 arrest by decreasing cyclin B1/Cdk1 complex activity. This regulation is achieved via direct p53-dependent repression of *cyclin B1* transcription [34] as well as by stabilization and nuclear retention of cyclin B1/Cdk1 complex [38]. Binding of p21 to cyclin B1/Cdk1 complex blocks its recruitment to centrosomes and activation by cdc25 family phosphatases [38] thus preventing the passage into mitosis and causing an arrest in G2 phase. This explains why the protein levels of cyclin B1 were stable at 3 and 6 hours and higher than non-irradiated cells. In fact, the upregulation of p21 at 3 hours in our model could have stabilized the G2/M checkpoint complex cyclin B1/Cdk1 and prevented the turnover of cyclin B1. This supports the slight arrest of irradiated Molt-4-LXSN cells in the G2/M phase after 24 hours only (unpublished data). Moreover, the G2/M arrest was accompanied by a significant increase in the percentage of Sub-G0 cells at 24 hours and 48 hours post-irradiation (unpublished data). In fact, in addition to its role in stabilizing G2/M checkpoints, p21 can also promote ceramide-induced apoptosis in hepatocarcinoma cells [39]. This may prove to be true in our model since p21 upregulation at 3 hours preceded the accumulation of ceramide at 14 hours and cell death at 24 hours and 48 hours.

## Conclusion

Based on previous studies and models, ceramide seems to play an important role in mediating both intrinsic and extrinsic apoptosis. The emerging data analysis suggests that irradiation triggers a p53-dependent response that involves both the extrinsic apoptotic pathway represented by Fas and TNFRSF10B receptors and the intrinsic apoptotic pathway represented by pro-apoptotic Bcl-2 family proteins, Bim, Bax and Noxa. The upregulation of these latter candidates precedes the accumulation of ceramide at 14 hours, which may place them upstream of it in both apoptotic signaling pathways. One can expect these candidates to exert a role either on the synthesis of ceramide or on its cellular localization. Therefore, future studies must be conducted in order to investigate if these candidates are in direct relation with ceramide and if ceramide is involved in mediating the extrinsic, intrinsic, or both apoptotic pathways triggered by irradiation in our model Molt-4-LXSN.

## Supporting information

S1 Fig Protein expression levels of altered genes in response to irradiation. Western blots of three independent experiments for the expression level of Noxa, Bim, Bax, Fas, p21, and Cyclin B1 were assayed on total lysates of Molt-4-LXSN cells after 3, 6, 8, 14 and 24 hours post-irradiation. GAPDH was used as loading control in membranes.
(TIF)

S1 Table Differentially expressed (DE) genes in Molt-4-LXSN cells at 3hrs vs. 0hr. A total of 105 DE genes identified at 3hrs as compared to time 0hr under the threshold of fold change (FC) of 2 or greater and significance less than 0.05 (82 upregulated and 23 downregulated, 3hrs vs. 0hr).
(XLSX)

S2 Table Differentially expressed (DE) genes in Molt-4-LXSN cells at 8hrs vs. 0hr. A total of 210 DE genes identified at 8hrs as compared to time 0hr under the threshold of fold change (FC) of 2 or greater and significance less than 0.05 (187 upregulated and 23 downregulated, 8hrs vs. 0hr).
(XLSX)

S3 Table Differentially expressed (DE) genes in Molt-4-E6 cells at 3hrs vs. 0hr. A total of 6 DE genes identified at 3hrs as compared to time 0hr under the threshold of fold change (FC) of 2 or greater and significance less than 0.05 (all 6 upregulated, 3hrs vs. 0hr).
(XLSX)

S4 Table Differentially expressed (DE) genes in Molt-4-E6 cells at 8hrs vs. 0hr. A total of 5 DE genes identified at 8hrs as compared to time 0hr under the threshold of fold change (FC) of 2 or greater and significance less than 0.05 (all 5 upregulated, 8hrs vs. 0hr).
(XLSX)

S5 Table List of unique and common differentially altered pathways in Molt-4-LXSN cells between 3hrs and 8hrs normalized to 0hr. Pathways listing of the unique pathways detected included 40 pathways (3hrs vs. 0hr) and 40 pathways (8hrs vs. 0hr). Pathways listing of the shared pathways detected included 60 pathways (3hrs vs. 8hrs vs. 0hr).
(XLSX)

S6 Table The expression matrix of the top 10 000 most variable probes of three independent experiments for Molt-4-LXSN_0h, Molt-4-LXSN_3h and Molt-4-LXSN_8h. (XLSX)

S7 Table Differentially expressed genes in Molt-4-LXSN cells at 3hrs vs. 0hr and their relation to some pathways. (XLSX)

S8 Table Differentially expressed genes in Molt-4-LXSN cells at 8hrs vs. 0hr and their relation to some pathways. (XLSX)

S9 Table Interaction analysis of the global molecular & biological processes in Molt-4-LXSN cells at 3hrs vs. 0hr. (XLSX)

S10 Table Interaction analysis of the global molecular & biological processes in Molt-4-LXSN cells at 8hrs vs. 0hr. (XLSX)

S11 Table Interaction analysis of some targeted proteins and cellular processes in Molt-4-LXSN cells at 3hrs vs. 0hr. (XLSX)

S12 Table Interaction analysis of some targeted proteins and cellular processes in Molt-4-LXSN cells at 8hrs vs. 0hr. (XLSX)
