# Supplementary figures and images for: Genomic alterations during p53-dependent apoptosis induced by γ-irradiation of Molt-4 leukemia cells
Source: PLoS One. 2017 Dec 22;12(12):e0190221. doi: 10.1371/journal.pone.0190221 (PMC5741252; doi:10.1371/journal.pone.0190221)

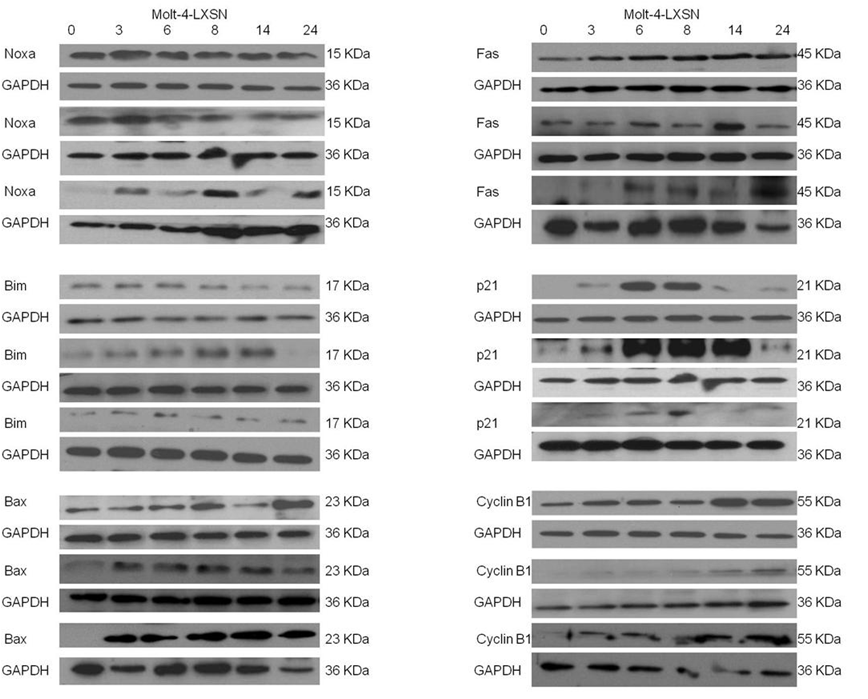

Supplement: S1 Fig — Western blots of three independent experiments for the expression level of Noxa, Bim, Bax, Fas, p21, and Cyclin B1 were assayed on total lysates of Molt-4-LXSN cells after 3, 6, 8, 14 and 24 hours post-irradiation. GAPDH was used as loading control in membranes. (TIF) [file pone.0190221.s001.tif]
